# Revealing the Introduction History and Phylogenetic Relationships of *Passiflora foetida sensu lato* in Australia

**DOI:** 10.3389/fpls.2021.651805

**Published:** 2021-07-29

**Authors:** Tara Hopley, Bruce L. Webber, S. Raghu, Louise Morin, Margaret Byrne

**Affiliations:** ^1^Biodiversity and Conservation Science, Department of Biodiversity, Conservation and Attractions, Kensington, WA, Australia; ^2^CSIRO Health & Biosecurity, Floreat, WA, Australia; ^3^School of Biological Sciences, The University of Western Australia, Crawley, WA, Australia; ^4^Western Australian Biodiversity Science Institute, Perth, WA, Australia; ^5^CSIRO Health & Biosecurity, Brisbane, QLD, Australia; ^6^CSIRO Health & Biosecurity, Canberra, ACT, Australia

**Keywords:** biocontrol, biogeography, classical biological control, invasion history, phylogeny, species introduction

## Abstract

Genomic analysis can be a valuable tool to assistmanagement of non-native invasive species, through determining source and number of introductions as well as clarifying phylogenetic relationships. Here, we used whole chloroplast sequencing to investigate the introduction history of *Passiflora foetida sensu lato* in Australia and clarify its relationship with other *Passiflora* species present. Phylogenetic analysis of chloroplast genome data identified three separate genetic lineages of *P*. *foetida s*. *l*. present in Australia, indicating multiple introductions. These lineages had affinities to samples from three separate areas within the native range in Central and South America that represented phylogenetically distinct lineages. These results provide a basis for a targeted search of the native range of *P*. *foetida s*. *l*. for candidate biological control agents that have co-evolved with this species and are thus better adapted to the lineages that are present in Australia. Results also indicated that the *Passiflora* species native to Australia are in a separate clade to that of *P*. *foetida s*. *l*. and other introduced *Passiflora* species cultivated in Australia. This knowledge is important to assess the likelihood of finding biological control agents for *P*. *foetida s*. *l*. that will be sufficiently host-specific for introduction in Australia. As *P*. *foetida s*. *l*. is a widespread non-native invasive species across many regions of the world, outcomes from this work highlight the importance of first evaluating the specific entities present in a country before the initiation of a biological control program.

## Introduction

Non-native invasive species can have significant negative impacts across their introduced range (Pyšek et al., 2012), transforming social, economic, and environmental values in managed and unmanaged landscapes (Pimentel, 2001; Pyšek et al., 2012; Simberloff et al., 2013; Marbuah et al., 2014). In many countries, such non-native species are second only to climate change as the biggest threat to native biological diversity (Vilà et al., 2011) and their impacts on agriculture amount to many billions of dollars annually (Oerke, 2006). Plant introductions may be unintended or deliberate, such as via horticultural trade or for pastoral improvement, and in many instances single taxa have been introduced on more than one occasion (Mack and Lonsdale, 2001; Cook and Dias, 2006). This variation in introduction history can influence the success or otherwise of introduced species (Kolar and Lodge, 2001) and impact the likelihood of progression along the invasion curve (Blackburn et al., 2011).

Recent advances in genomic analysis is enabling more sophisticated investigations of the invasion history and colonization dynamics of introduced species (Richards et al., 2006; Estoup and Guillemaud, 2010; Cristescu, 2015). New technology provides the level of molecular resolution necessary to resolve complex demographic events associated with non-native invasions (Barrett et al., 2008; Cristescu, 2015). Such molecular data can provide answers to specific questions such as: how often and how many lineages of a non-native invasive species have been introduced to the new range (Nissen et al., 1995); what are the source(s) of these introductions (Hanlon et al., 2000; Burrell et al., 2015; Chown et al., 2015); what is the phylogenetic relationship between the introduced lineages and any other co-occurring related species (Hanlon et al., 2000); and is there any evolutionary divergence between populations of the focal species in the native and introduced ranges (Nissen et al., 1995; Vandepitte et al., 2014; Burrell et al., 2015; Chown et al., 2015; Ferrero et al., 2015).

There is an overall trend of reduction in genetic diversity for most invasive plant species during introduction (Dlugosch and Parker, 2008), although there have been rare cases of successful invaders that have shown higher levels of within population genetic diversity in the introduced range than in the native range (Novak and Mack, 1993; Genton et al., 2005). Theory would suggest that a small number of individuals introduced to a new area would lead to founder effects and a low level of genetic diversity (Nei et al., 1975). Selection for preadapted genotypes after introduction may also lead to a reduction in diversity (Mack et al., 2000). In contrast, the introduction of a large number of individuals to a new region and/or multiple introductions from different sources have been shown to lead to higher levels of genetic diversity in the introduced range (Dlugosch and Parker, 2008; Pairon et al., 2010).

Identifying the source(s) of invasive plants is particularly useful for refining biosecurity protocols and to inform classical biological control (hereafter biocontrol) programs. Knowing the origin of introductions can be used to mitigate risk and refine border biosecurity arrangements (Maebara et al., 2020; Ricciardi et al., 2020). The same knowledge can also be used as a primary step to prioritize where to search for potential suitable, co-evolved biocontrol agents in the native range (Goolsby et al., 2006b; Gaskin et al., 2013; Kwong et al., 2017; McCulloch et al., 2020). Narrowing this search area is particularly relevant for target species with a broad geographic range or climatic niche.

Biocontrol programs also benefit from clarifying the taxonomy of the target invasive plant and determining its phylogenetic relationships with closely related species and the presence of any hybridization (Gaskin, 2003; Gaskin et al., 2013; McCulloch et al., 2020). Plant species that are phylogenetically more distant to the target plant are less likely to be suitable hosts for the same specialized natural enemies (Gilbert et al., 2012). Therefore, a detailed understanding of the phylogeny of the target and its congeners that are present in the introduced range, whether they are native or cultivated, enables predictions on the likelihood of finding biocontrol agents that will be sufficiently specific for introduction. This information is also used to guide development of the list of non-target plant species, following the centrifugal phylogenetic method (Wapshere, 1974; Briese, 2003), to use in host-specificity tests with the candidate biocontrol agents in order to assess the risks they could pose.

Knowledge of the different genetic lineages of the invasive plant present in the introduced range can improve management outcomes. Such variation can be due to the type of introduction, either as a result of a mixed introduction or multiple introductions. Variability can also be due to post-introduction selection or adaptive change. Rapid evolution due to strong selection pressures has been shown in some introduced plant populations (Oduor et al., 2011; Turner et al., 2014). Many successful invasive species also have traits such as a fast reproductive cycle or rapid maturity, that promote or enhance the opportunities for adaptation (Baker, 1974; Sakai et al., 2001). Comparing genetic variation of the plant between populations in the native and introduced ranges and identifying genetic lineages can assist with the selection of better adapted candidate biocontrol agents that are more likely to be effective (Moody et al., 2016; Kwong et al., 2017; Gaskin et al., 2019; Morin, 2020). Understanding the spatial distribution of distinct genetic entities can also inform management design for improved control, such as where buffer zones are positioned to avoid different variants moving to new areas (Moore et al., 2008).

Despite the clear advantages that molecular insights provide for management of invasive plants, a robust understanding of introduction history, genetic diversity and taxonomic identity remains poorly known for many of the world’s most widespread and threatening species. *Passiflora foetida* L. *sensu lato* (Passifloraceae) is an example of a threatening invader that has been widely introduced across many regions of the world, including South-East Asia, South Pacific Islands, China, Hawaii, India, Madagascar, and Australia (Yockteng et al., 2011). It is a fast-growing vine native to Central and South America that climbs over vegetation, including trees, and smothers plant communities (Lohr et al., 2016; Jucker et al., 2020). Traditional methods of control, including manual removal and herbicides, are not practical or cost-effective, particularly in situations where *P*. *foetida s*. *l*. infestations are in remote regions that are challenging to access (Jucker et al., 2020). Because of the extent of its distribution and severity of negative impacts it causes to environmental and socioeconomic values, *P*. *foetida s*. *l*. is being considered as a target for biocontrol in Australia (Webber et al., 2014; Scott et al., 2018).

The *Passiflora* genus is extensive, with more than 560 species recognized (Krosnick et al., 2013). Many species of *Passiflora* are also used around the world commercially as either horticultural crops (passionfruit) or as ornamental plants, including hybrids and varieties, and a number of countries where *P*. *foetida s*. *l*. has been introduced also have native *Passiflora* species (Vanderplank, 2000). Whilst consistently placed in subgenus *Passiflora* section *Dysosmia* DC, the species level taxonomy of *P*. *foetida* specifically, and multiple other taxa within section *Dysosmia* more generally, appears to be uncertain and problematic, with no molecular analyses available to confirm recent species circumscriptions based on morphology alone (Vanderplank, 2013; Svoboda, 2018; Svoboda and Ballard, 2018).

In Australia, where *P*. *foetida s*. *l*. has invaded across the northern extent of the continent, the earliest known record of introduction dates to 1854 (Anon, 1854), with concern already being raised about its invasive nature by 1892 (Holtze, 1892). However, little is known about the introduction history before or after those events. In recent decades the vine has become a major invader that can transform ecosystems (Beard et al., 1984; Kirkpatrick et al., 1988; Russell-Smith and Lee, 1992; Cowie and Werner, 1993; Preece et al., 2010; Lohr et al., 2016), and has been recognized as one of the weed species that poses the greatest threat to biodiversity on the islands of the Pilbara and Kimberley regions of Western Australia (Lohr et al., 2015, 2016). In addition to *P*. *foetida s*. *l*., at least 59 other *Passiflora* taxa have been introduced into Australia for horticultural and ornamental purposes (Randall, 2007). Several of these species have also become naturalized and invasive after escaping from cultivation (Randall, 2007). There are also six *Passiflora* taxa that are native to Australia (*P. aurantia* var. *aurantia* G.Forst., *P. aurantia* var. *pubescens* Bailey, *P. aurantioides* (K.Schum.) Krosnick, *P. cinnabarina* Lindl., *P. kuranda* Krosnick & A.J.Ford, *P. herbertiana* Ker Gawl.). Taken together, this set of circumstances makes *P. foetida s*. *l*. in Australia an ideal target for incorporating a molecular approach to addressing knowledge gaps to facilitate development of novel management solutions to improve control outcomes.

Here we use a whole chloroplast sequencing approach to assess the phylogenetic relationship between multiples accessions of *P*. *foetida s*. *l*. in order to identify the lineage(s) present in the introduced range in Australia and their most likely source area(s) in the native range in Central and South America. We also use the same approach to confirm the phylogenetic position of *P*. *foetida s*. *l*. amongst all taxa of *Passiflora* - native and non-native (including both commercial varieties and naturalized species) - that are present in Australia. Specifically, we sought (1) to determine whether the presence of *P*. *foetida s*. *l*. in Australia is the result of a single or multiple introductions; (2) to identify the source of the introduction(s) and any evolutionary divergence between the native range and Australian range; and (3) to determine the phylogenetic relationship between the introduced lineages and any other co-occurring *Passiflora* species. Our study demonstrates how the application of advanced molecular tools to explore the introduction history of non-native invasive plants can inform control programs. We discuss the implications of these results for the biocontrol program targeting *P*. *foetida s*. *l*. in Australia and frame these findings for the broader application of biocontrol for this threatening invader in other parts of its non-native range.

## Materials and Methods

### Focal Taxon

The most recent circumscriptions of section *Dysosmia*, to which *Passiflora foetida s. l.* belongs, recognize approximately 30 taxa (Vanderplank, 2013; Svoboda, 2019), including two major species complexes: *P. foetida* L. and *P. ciliata* Aiton. The taxonomy of this section has been problematic for over 200 years (Svoboda and Ballard, 2018) due to extreme variation in morphological characters and no molecular phylogeny to date (Vanderplank, 2013; Svoboda, 2018, 2019; Svoboda and Ballard, 2018). Moreover, there is evidence of hybridization between taxa after introduction into areas with co-occurring (native or introduced) and closely related taxa (Vanderplank, 2013).

Over c. 170 years, a number of names have been applied to *Passiflora* section *Dysosmia* collections from non-native populations across Australia, including *Passiflora foetida* L., *Passiflora foetida* L. *var. hispida* (DC ex Triana & Planchon) Killip ex Gleason (*syn. Passiflora vesicaria* L. *var. vesicaria*), *Passiflora foetida* L. *var. ellisonii* Vanderpl., and *Passiflora foetida* L. *var foetida* (Vanderplank, 2013; Svoboda et al., 2016; Atlas of Living Australia, 2021). An examination of available material from the Australian range suggests that there remains considerable uncertainty as to the validity of many of these determinations (Webber, unpublished data; Ohlsen, 2020).

For the focal taxon in this study (both existing herbarium samples as well as new material), characters used to select material included pubescent shallow tri-lobed leaves, glandular hairs on all vegetative parts, and ornate pinnatisect floral bracts with glandular tips. Flowers and ripe fruits were rarely available to confirm other useful diagnostic characters. It is recognized that many of these vegetative characters also fit other taxa within section *Dysosmia* (Vanderplank, 2013), but are, in turn, unique to *Dysosmia* within the broader Passifloraceae (Svoboda, 2019). Resolution of this taxonomic uncertainty is well beyond the scope of this study. For the purposes of this work we refer to the entity(ies) present in Australia as *P. foetida* s. l. following the recommended national convention established by Ohlsen (2020).

It remains unclear whether the placement of section *Dysosmia* within subgenus *Passiflora* is appropriate. Recent evidence suggests that subgenus *Passiflora* is paraphyletic (Ramaiya et al., 2014; Silva and Souza, 2020; but see Muschner et al., 2012; Krosnick et al., 2013), aligning with the earlier work of Yockteng and Nadot (2004) that suggested *Dysosmia* should be recognized at the subgenus level separate from subgenus *Passiflora*. Other *Passiflora* taxa known to occur in Australia and prioritized for inclusion in this study include those introduced for commercial and horticultural reasons (largely restricted to section *Granadilla*, subgenus *Passiflora*; Muschner et al., 2012), as well as six species native to Australia (subgenus *Decaloba;* Krosnick et al., 2009, 2013).

**FIGURE 1:**
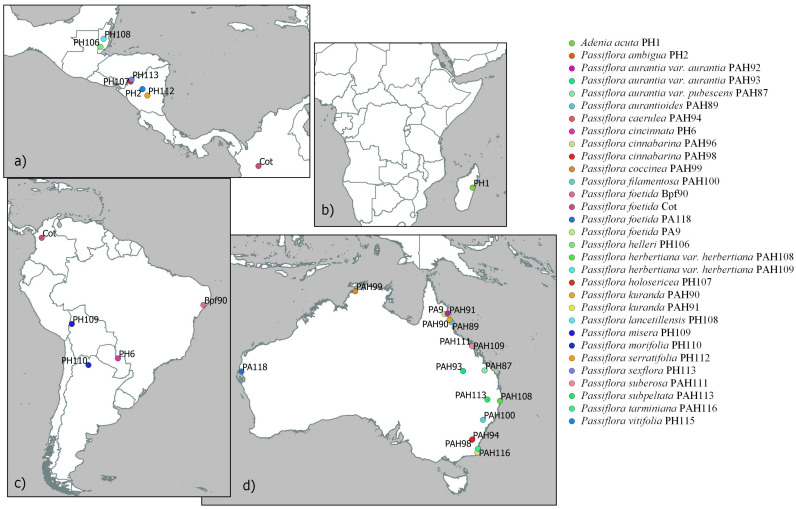
Geographic location (when known) of non-cultivated collections of *Passiflora* species and *Passiflora foetida sensu lato* samples in the “Passifloraceae” dataset in **(a)** Central America, **(b)** Madagascar, **(c)** South America, and **(d)** Australia.

*Sample sourcing* A wide geographic range of samples of *P*. *foetida s*. *l*., other *Passiflora* species known to occur in Australia and one species in a different genus in Passifloraceae were used for this study (Figures 1, 3). These samples included 13 of the 59 species that have been introduced into Australia and represent those that are most invasive or often used in cultivation. Leaf samples were obtained either from herbarium specimens or from fresh material from both natural and managed populations. A total of 192 samples were sent for sequencing, however, due to low quality DNA and low quantities of DNA, particularly from the herbarium material, there were a number of failures and these were removed before analysis.

Herbarium specimens contributed 89 samples to the final analysis (Supplementary Table 1). These included 72 representative specimens held at the Missouri Botanic Gardens (MOBOT) determined as *P*. *foetida s*. *l*. from the native range, as well as two other species in Passifloraceae for use as outgroups, and thirteen specimens from the Australian National Herbarium (CANB) and four specimens from the Queensland Herbarium (QLD) representing native and non-native *Passiflora* species in Australia (Supplementary Table 1). Small amounts of leaf material were taken from each of the herbarium specimens and stored on silica gel until DNA was extracted. Genomic DNA was extracted using the protocol outlined in Lade et al. (2014) with minor modifications; samples were ground using a Qiagen TissueLyser, DNA was left to precipitate in ethanol for 48 h, and a wash step with 70% ethanol was added to the end of the protocol.

Samples taken from live plants in the field from a variety of sources and subsequently dried (silica gel) contributed a further 54 samples to the final analysis (Supplementary Table 1). Fresh leaf samples were collected from plants determined as *P*. *foetida s*. *l*. at 36 locations across Australia, a single plant sampled at each location for this work. A total of 8 samples of *Passiflora* species currently in cultivation in Australia were obtained from either nursery stock or plant breeders (Supplementary Table 1). A number of DNA samples were also provided by collaborators, including a single sample from a cultivated *P. subpeltata* Ortega plant in Australia, a single sample of *P*. *foetida s*. *l*. collected in Malaysia and four samples of *P*. *foetida s*. *l*. collected in each of Colombia and Brazil (Supplementary Table 1). All collected fresh samples were stored on silica gel before DNA extraction using a modified CTAB method (Doyle and Doyle, 1987), with the addition of sodium sulfite (Byrne et al., 2001) and 1% w/v polyvinylpyrrolidone to the extraction buffer.

### Sequencing and Analysis

Library preparation was undertaken by the Australian Genome Research Facility (AGRF) using either the Illumina Truseq Nano gDNA shotgun protocol or the NEBnext Ultra 2 library preparation. Sequencing was conducted on the HiSeq2500 or NovaSeq 6000 platforms. Raw paired-end sequences were imported to GENEIOUS 11.1.5^1^ (Kearse et al., 2012), and the map to reference tool was used to trim and map sequences using the medium-low sensitivity with five iterations. The *P*. *foetida s*. *l*. samples were mapped to the *P. foetida* complete chloroplast genome (MK694932.1) and all other samples were mapped to the *Passiflora edulis* Sims complete chloroplast genome (MF807938.1), downloaded from Genbank. The mean coverage across samples was 628, with coverage ranging from 27 to 2386×, samples with mean coverage below 25 were considered failures, only 7 samples had mean coverages below 50 and these were visually inspected to make sure that there was coverage across the gene regions that were subsequently used. A consensus sequence was generated from the mapped reads with the highest quality setting and then annotated from the reference sequence with an 85% similarity for *P*. *foetida s*. *l*. and a 70% similarity for the other *Passiflora* species.

The data were split into two groups, one dataset containing 117 individuals, all the samples of *P*. *foetida s*. *l*. plus *Passiflora ambigua* Hemsl. as an outgroup (hereafter referred to as the ‘*foetida* dataset’) and another containing 31 samples, all the other Passifloraceae species and a few representative *P*. *foetida s*. *l*. samples (hereafter referred to as the “*Passifloraceae* dataset”), with *Adenia acuta* W.J. de Wilde as an outgroup. Genes were extracted from the individual consensus sequences for each dataset; 107 genes for the *foetida* dataset and 73 genes for the *Passifloraceae* dataset (Supplementary Table 2). The extracted gene sequences were aligned and then concatenated. The concatenated sequences were partitioned into genes and run through MODELTEST-NG to determine the optimal model of molecular evolution and gamma rate heterogeneity for subsequent use in RAxML-NG and MRBAYES (Supplementary Table 2). The best-fitting models were determined according to the Akaike Information Criterion (Akaike, 1974).

Maximum likelihood analysis was performed using RAxML-NG v.0.6.0 (Kozlov et al., 2019) with partitioned PHYLIP files using the appropriate model for each gene/partition from MODELTEST-NG. The maximum likelihood (ML) tree search used 10 randomized parsimony starting trees and auto MRE bootstrapping convergence criteria with a maximum of 1500 replicates and a cut-off value of 0.03.

Bayesian inference was performed using MRBAYES v3.2.6 with the partitioned datasets using the appropriate model for each partition/gene determined in MODELTEST-NG. Posterior probabilities were generated from 1 × 10^7^ generations, sampling at every 1000th iteration, and the analysis was run three times with one cold and three incrementally heated Metropolis-coupled Monte Carlo Markov chains, starting from random trees. The adequacy of run lengths was assessed in TRACER 1.7.1 (Rambaut et al., 2018) using the effective sample size (ESS) parameter and convergence based on comparison of the independent MCMC runs. Trees were visualized using FIGTREE v.1.4.3^2^.

Samples from the *foetida* dataset were split into two groups, native range samples and Australian samples, and nucleotide diversity (π) was estimated using the R packages APE v 5.3 (Paradis and Schliep, 2018) and PEGAS v 0.12 (Paradis, 2010) in R 3.6.1 (R Core Team, 2019). The average number of nucleotide differences between groups was estimated using DNASP 5.10.01 (Librado and Rozas, 2009). An assessment of genetic variation among lineages in the *foetida* dataset was determined using Analyses of Molecular Variation (AMOVA), as implemented in the program ARLEQUIN 3.5.2.2 (Excoffier and Lischer, 2010).

## Results

**FIGURE 2:**
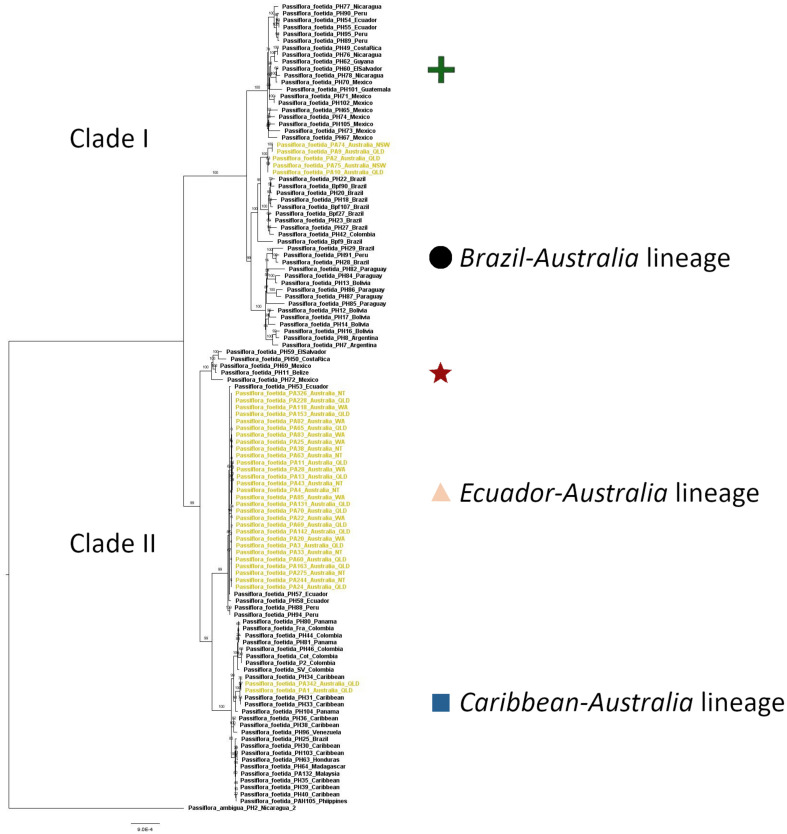
Phylogenetic tree of samples in the *foetida* dataset (all samples of *Passiflora foetida sensu lato* plus *Passiflora ambigua* as an outgroup) based on the maximum likelihood analysis, shown with bootstrap confidence values. The samples from Australia are colored in khaki. Each of the clades are assigned a symbol which matches those used in the map presented in Figure 3.

**FIGURE 3:**
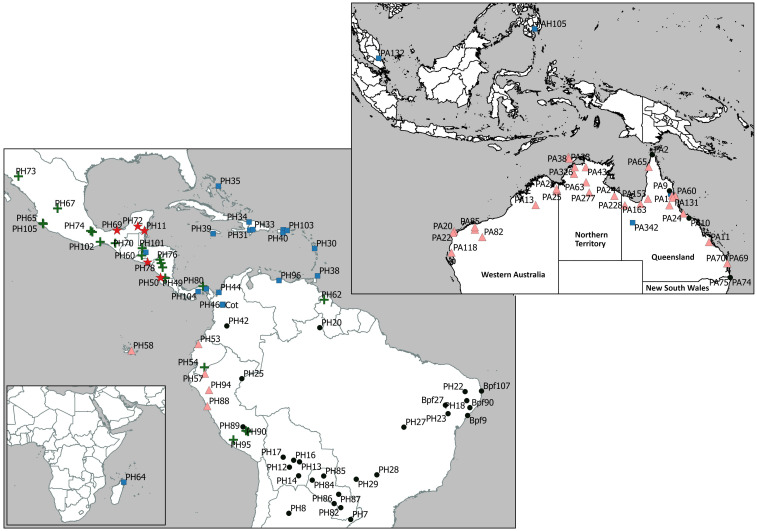
Geographic location of *Passiflora foetida sensu lato* samples used in this study (*foetida* dataset), from the native range in Central and South America and introduced range in Australia, South-East Asia and Africa (Madagascar), grouped according to the five sub-clades (each identified with a different symbol) in the phylogenetic tree shown in Figure 2.

The *foetida* dataset was used to identify the relationship of *P*. *foetida s*. *l*. samples from the introduced range, primarily Australia, to samples from the native range in Central and South America and gave an alignment of 73,066 sites representing 107 genes, with no missing data. Both the maximum likelihood (Figure 2) and Bayesian analysis (Supplementary Figure 1) recovered the same tree topology with highly resolved clades and well supported branches. The topology of the phylogenetic tree generated using maximum likelihood analysis (Figure 2) showed two main clades (I and II) with several lineages within each. The samples of *P*. *foetida s*. *l*. from Australia were positioned in three places in the tree. The majority of samples collected from locations in the Australian regions of Western Australia, Northern Territory and some of the locations in Queensland clustered with samples from Ecuador and Peru (the *Ecuador-Australia* lineage; Figures 2, 3) in Clade II. Two samples from locations in Queensland clustered with all samples from the Caribbean and some from Central America and the northern part of South America (the *Caribbean-Australia* lineage; Figures 2, 3), along with samples from South-East Asia and Africa, within Clade II. Several Australian samples from Queensland and New South Wales clustered with samples from Brazil and one from Colombia (the *Brazil-Australia* lineage; Figures 2, 3) in Clade I that comprises samples from across South America.

The genetic differences in the tree showed a minimum percentage sequence identity of 98.308% in the alignments between the outgroup *P. ambigua* and a *P*. *foetida s*. *l*. sample from Mexico (PH73). Amongst all *P*. *foetida s*. *l*. samples the greatest difference across 73,066 sites was across lineages, with a percentage sequence identity of 99.341% between samples PH95 (Peru) and PH44 (Colombia). Within samples of *P*. *foetida s*. *l*. from Australia, the greatest difference was a percentage sequence identity of 99.465% between individuals in the separate lineages (i.e., between sample PA9 and samples PA1). Sequence differences were lower within lineages; within the *Ecuador-Australia* lineage there were between 0 and 26 base pair differences, and there were a similar number of differences within the *Brazil-Australia* (1–23) and *Caribbean-Australia* (10) lineages.

The average percentage sequence identity between the Australian samples in the *Brazil-Australia* lineage and those samples from Brazil in the sub-clade was 99.897%. Less differentiation was found in the other lineages, with average percentage sequence identity between the Australian samples in the *Caribbean-Australia* lineage and those samples from the Caribbean in the sub-clade was 99.967%, and between the Australian samples in the *Ecuador-Australia* lineage and those samples from Ecuador and Peru was 99.991%. These values are all smaller than the level of differentiation between the three lineages represented by samples collected in Australia. Nucleotide diversity (π) was found to be 0.0011 (variance = 2.7 e-07) for the 37 *P*. *foetida s*. *l*. samples from the introduced range in Australia and 0.0027 (variance = 1.7 e-06) for the 79 samples of *P*. *foetida s*. *l*. from the native range in Central and South America. Among-group genetic variation estimates from AMOVA analyses were found to be significant (P = 0.057) with 82% of variation among lineages.

**FIGURE 4:**
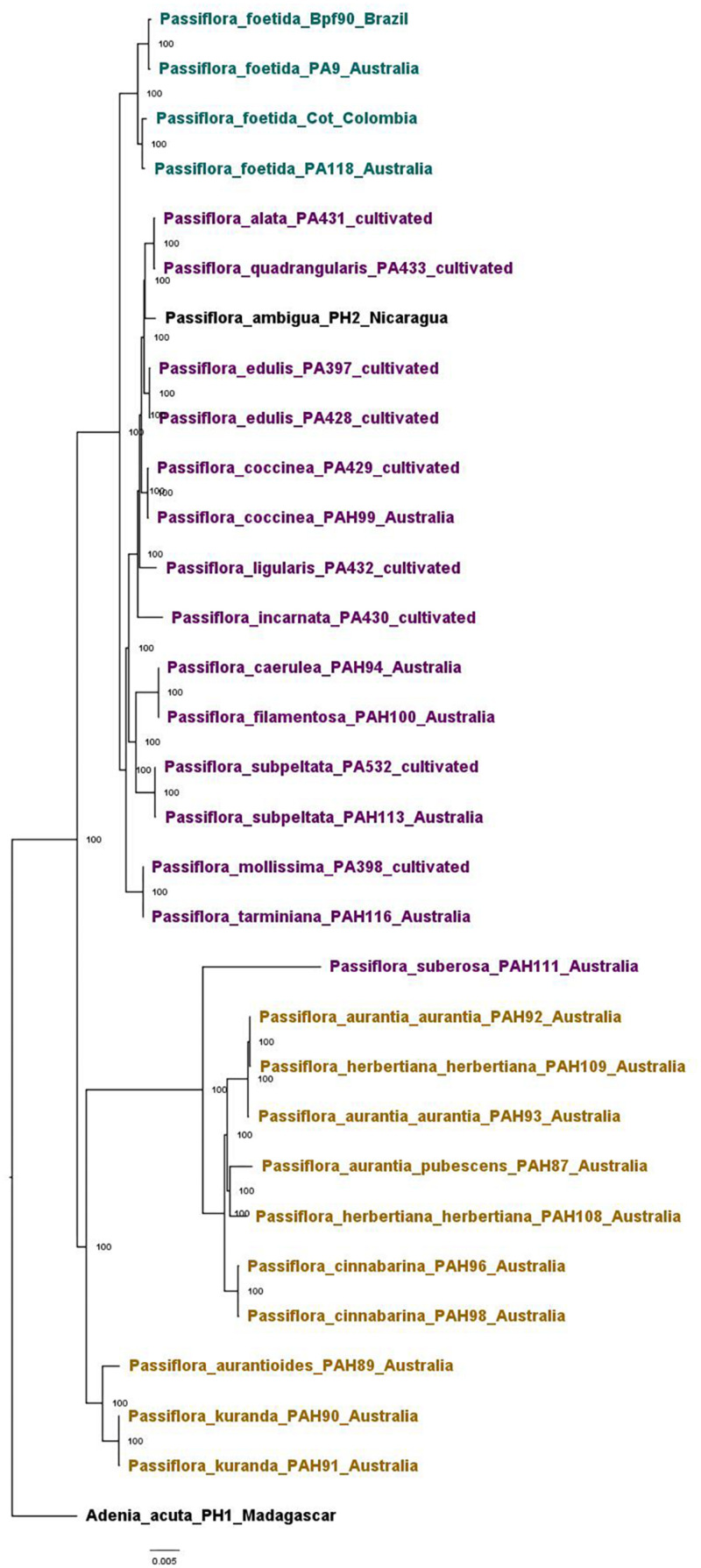
Phylogenetic tree of samples in the *Passifloraceae* dataset based on the Bayesian analysis, shown with posterior probabilities values. Samples of the native *Passiflora* species from Australia are colored in khaki, the non-native species in Australia (both cultivated and invasive) in purple, and *Passiflora foetida sensu lato* in teal. Samples used as outgroups across analyses are in black.

The *Passifloraceae* dataset was used to confirm the phylogenetic position of *P*. *foetida s*. *l*. amongst *Passiflora* species native to Australia, as well other *Passiflora* species introduced to Australia and those used commercially. It gave an alignment of 68,697 sites representing 73 genes, with 0.03% missing data. The same tree topology was obtained with both the maximum likelihood (Supplementary Figure 2) and Bayesian analysis and trees were highly resolved and well supported. The topology of the phylogenetic tree generated using Bayesian analysis (Figure 4) showed two main clades, one containing the Australian native *Passiflora* species along with one sample of the non-native *Passiflora suberosa* L. from Australia, and the other containing representative *P*. *foetida s*. *l*. samples, the cultivated *Passiflora* species and all other non-native *Passiflora* species. The samples of *P*. *foetida s*. *l*. were clustered in a separate sub-clade from the cultivated and other non-native *Passiflora* species samples from Australia. The Australian native species were clustered into two separate sub-clades.

## Discussion

Our analysis of relationships among introduced and native range samples of *P*. *foetida s*. *l*. and its relatives demonstrates the value of genetic analysis of non-native invasive species for determining geographic origins, including providing evidence for multiple introductions (Nissen et al., 1995; Hanlon et al., 2000; Chown et al., 2015). The genomic analysis identified three separate genetic lineages of *P*. *foetida s*. *l*. in Australia with affinities to three separate areas within the native range. The dominant lineage was present in samples from across northern Australia and was found to be closely related to samples from locations in Ecuador and Peru. The other two lineages had restricted distributions in localized areas in Queensland and New South Wales and were closely related to samples from Brazil and from the Caribbean, respectively. Our analysis also demonstrated that *P*. *foetida s*. *l*. in Australia is genetically differentiated from related *Passiflora* species native to Australia and from cultivated species used in Australian horticulture. This is essential foundational information indicating that there are reasonable prospects for finding biocontrol agents specific to *P*. *foetida s*. *l*. Finding suitable agents would be more challenging if *P*. *foetida s*. *l*. had been found to be more closely related to the cultivated and/or native *Passiflora* species present in Australia (e.g. Lesieur et al., 2020).

The presence of three separate lineages of *P*. *foetida s*. *l*. in Australia with likely sources from geographically distinct regions in the native range is indicative of multiple introductions. Introductions of species to new areas often involves limited propagules leading to founder effects that produce bottlenecks reducing genetic variation (Lawson Handley et al., 2011). Multiple introductions can alleviate founder effects and increase genetic variation, which has been reported to facilitate population growth and invasiveness (Petit et al., 2004; Dlugosch and Hays, 2008). Our study adds to evidence from other studies that have identified patterns of multiple introductions in invasive plant species using a genomic approach. For example, genomic analysis of the highly invasive *Imperata cylindrica* (L.) P.Beauv. in south-eastern United States (Burrell et al., 2015) and of *Oxalis pes-caprae* (L.) in the Mediterranean (Ferrero et al., 2015) clearly showed that multiple introductions occurred from their respective native range in Japan (as well as from a related species in Brazil) and South Africa.

Anthropogenic movement of plants between regions often results in low genetic diversity in introduced populations, especially when the introductions are accidental and where they occur across continents. For example, the introduced populations of *O*. *pes-caprae* in the Mediterranean were found to be less genetically diverse than populations in the native range (Ferrero et al., 2015). Our results are consistent with this general observation as the samples of *P*. *foetida s*. *l*. from the introduced range in Australia showed lower nucleotide diversity than the samples from the native range in Central and South America. Whilst the reduced diversity in Australia may be due to fewer samples taken from this region, these samples were representative of three of the lineages found in the dataset. This suggests there has been genetic bottlenecks and resulting reduction in chloroplast diversity through the introduction process. The plastome of *Passiflora* generally has uniparental inheritance, however, in rare cases biparental inheritance has been observed, generally caused by hybridization between species with plastome size variation (Shrestha et al., 2021). Uniparental inheritance is beneficial for tracking source of introductions as it takes longer to show signs of lineage mixing. An assessment of the nuclear genome would be more likely to identify increased genetic diversity that could be expected due to contemporary mixing between lineages.

Knowing the likely source locations of an introduced species is important for guiding the search for candidate biocontrol agents that are highly compatible with the target weed, particularly for a species like *P*. *foetida s*. *l*. with uncertain taxonomy and a geographic range as circumscribed that encompasses a broad climatic niche (Goolsby et al., 2006a; Hawkins et al., 2007; Ndlovu et al., 2013; Bell et al., 2014). Based on our results, Ecuador and Peru should be regarded a priority area to search for candidate agents for *P. foetida s. l.*, especially biotrophic fungal pathogens that are typically highly specialized, since this is the likely provenance of the dominant, widespread lineage that exists in Australia. More extensive sampling of *P. foetida s. l.* in the native range could reveal other areas where this dominant lineage occurs. Whether or not efforts should be made to extend searches for candidate agents in other areas of the native range where the secondary lineages originate from remains an open question that would benefit from being informed by complementary ecological studies. Circumstantial evidence from field observations suggests that these secondary lineages of *P*. *foetida s*. *l*. present on the eastern Australian coast are less prevalent in the landscape than the dominant lineage found across Western Australia, the Northern Territory and drier parts of western Queensland. Such differences in abundance and distribution could be explained by a difference in relative competitive ability of the lineages present in contrasting climates (the east coast populations are exposed to less severe dry seasons and more consistent precipitation), or by the genetic differences revealed in this study. Whilst uniparental inheritance of chloroplast data has been useful in elucidating the source of the introductions, it has limitations for evaluating contemporary population dynamics of invasive lineages. Further work elucidating variation of the nuclear genome within each of the different chloroplast lineages identified in Australia will be needed to determine if hybridization among *P*. *foetida s*. *l*. lineages from different geographical source areas has occurred and to characterize current patterns of gene flow across Australia. Given the short generation times of *P*. *foetida s*. *l*. and generalist volant-mediated dispersal (including birds and flying foxes; Palmer et al., 2000; Whittaker and Jones, 2006; Preece et al., 2010), the opportunity for long distance gene flow is considerable.

The *Passiflora* genus contains a large amount of diversity with over 560 currently described species (Krosnick et al., 2013). The phylogeny of *Passiflora* species obtained in our study is consistent with previous systematic work on the genus based on a single gene (Yockteng and Nadot, 2004). Our results showed that the native *Passiflora* species in Australia (found in subgenus *Decaloba*) are in a separate clade to the non-native *P*. *foetida s*. *l*. (found in subgenus *Passiflora* section *Dysosmia*; but see Silva and Souza, 2020) and other *Passiflora* species cultivated in Australia (found primarily in subgenus *Passiflora* section Granadilla). The clear phylogenetic distance between *P*. *foetida s*. *l*. and the native species in Australia means that it may be possible to find natural enemies of *P*. *foetida s*. *l*. in its native range that are unlikely to impact native *Passiflora* species in Australia and thus promising for biocontrol. While the majority of *Passiflora* species in cultivation are relatively more related at the subgenus level to *P*. *foetida s*. *l*., enough phylogenetic separation was observed to provide reasonable confidence that biocontrol agent(s) with sufficient specificity and minimal off-target impacts may be found.

Whilst this work has provided insight into the introduction history of *P. foetida s*. *l*. in Australia, the taxon is also widely introduced elsewhere in the world, including South-East Asia, on South Pacific Islands, and in China, Hawaii, India, and Madagascar (Yockteng et al., 2011). Given this global invasion footprint, any progress towards a biocontrol solution that is made in Australia may well have relevance for controlling invasions elsewhere. However, given our findings identifying multiple genetic lineages of *P*. *foetida s*. *l*. and overlapping range between lineages present in Australia, any control efforts looking to apply this work elsewhere should first evaluate the specific lineages present before the initiation of biocontrol programs. Whether or not the genetic variation observed between the three lineages of *P. foetida s. l.* in Australia will cause problems for the development of bio control agents, particularly for some fungal pathogens that are known to be highly specific, remains a priority for further research. Moreover, given the variation in genetic diversity uncovered in samples from eastern Australia as part of this study, as well as previous observations that species in section *Dysosmia* readily hybridize (Vanderplank, 2013), a more thorough investigation of morphological and molecular diversity of *P*. *foetida s*. *l*. across native and introduced regions is recommended to produce a more robust taxonomic delimitation for the complex (Svoboda, 2015). In providing clarity for the introduction history of *P. foetida s. l.* in Australia, our study adds to the growing body of work demonstrating the benefit of applying genomic approaches to identify key components of the introduction history of non-native plant species. For those taxa that represent key threats to environmental and socioeconomic values, using this information to make predictions on the prospects for biocontrol and improve the development of control programs will help deliver more effective management strategies.

## Data Availability Statement

The datasets presented in this study can be found in online repositories. The names of the repository/repositories and accession number(s) can be found below: https://www.ebi.ac.uk/ena/browser/view/PRJEB43378, https://figshare.com/articles/dataset/Passiflora_genomic_datasets/13352351/1.

## Author Contributions

MB, BW, SR, and LM developed the project. TH and MB designed the study. SR and LM sourced most samples. TH did genetic data analysis and drafted the manuscript with input from MB and BW. All authors contributed to revision of the manuscript.

## Conflict of Interest

The authors declare that the research was conducted in the absence of any commercial or financial relationships that could be construed as a potential conflict of interest.

## Publisher’s Note

All claims expressed in this article are solely those of the authors and do not necessarily represent those of their affiliated organizations, or those of the publisher, the editors and the reviewers. Any product that may be evaluated in this article, or claim that may be made by its manufacturer, is not guaranteed or endorsed by the publisher.

## Footnotes

1
https://www.geneious.com

2
http://tree.bio.ed.ac.uk/software/figtree/
