# Supplementary figures and images for: Revealing the Introduction History and Phylogenetic Relationships of Passiflora foetida sensu lato in Australia
Source: Front Plant Sci. 2021 Jul 29;12:651805. doi: 10.3389/fpls.2021.651805 (PMC8358147; doi:10.3389/fpls.2021.651805)

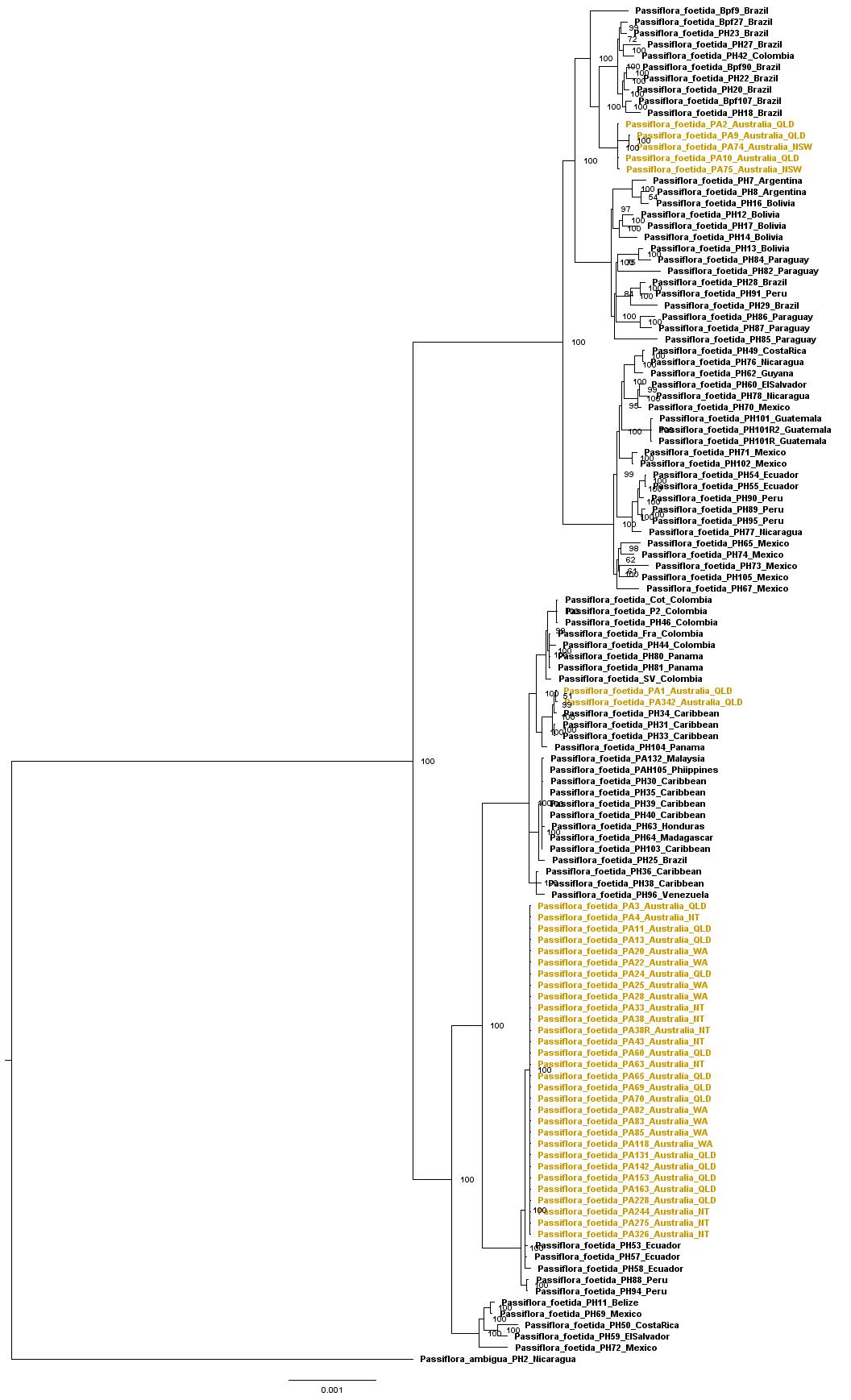

Supplement: Supplementary file 1 [file Image_1.jpg]

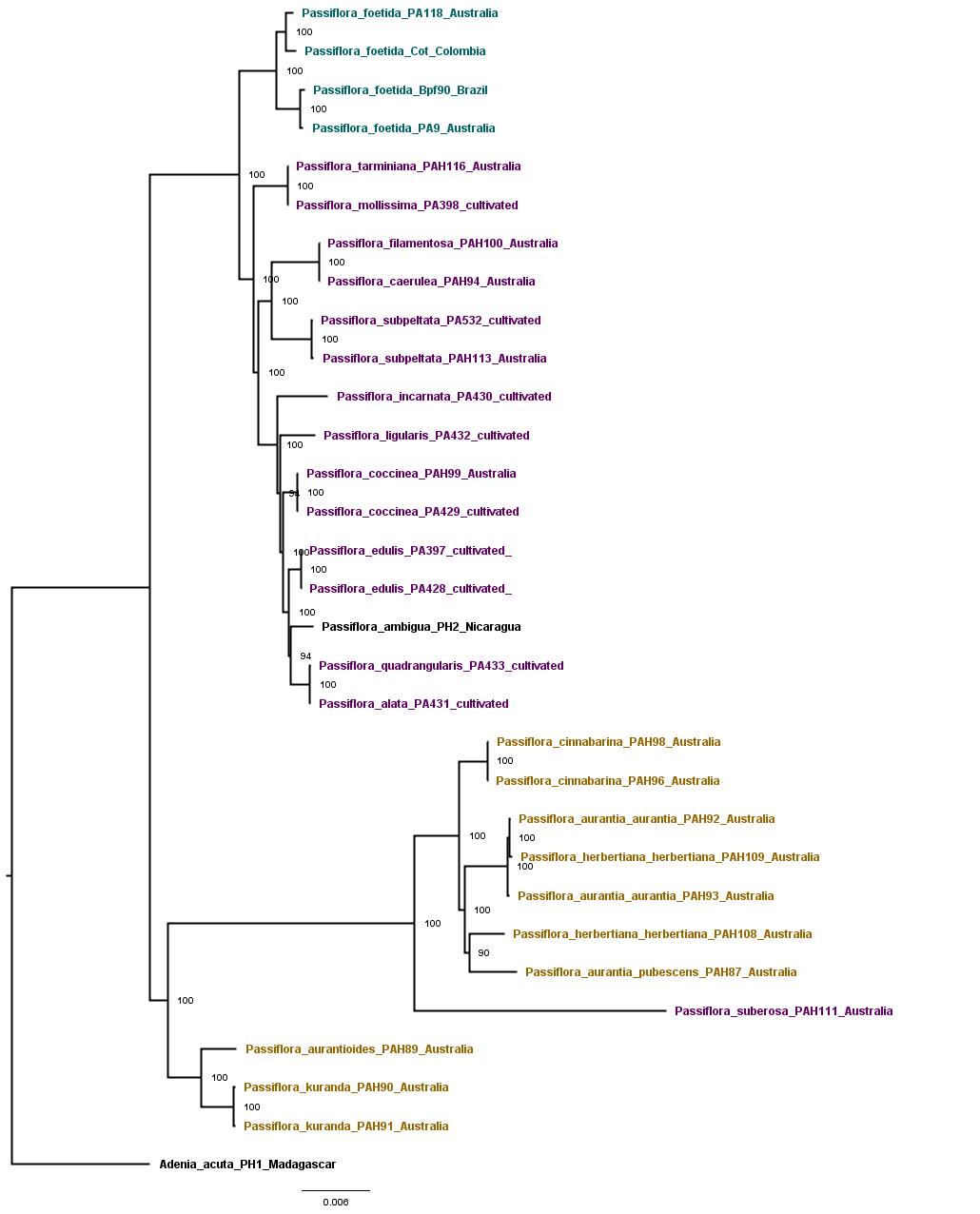

Supplement: Supplementary file 2 [file Image_2.jpg]
