# Supplementary material for: Revealing the Introduction History and Phylogenetic Relationships of Passiflora foetida sensu lato in Australia
Source: Front Plant Sci. 2021 Jul 29;12:651805. doi: 10.3389/fpls.2021.651805 (PMC8358147; doi:10.3389/fpls.2021.651805)
Supplement: Supplementary file 5 [file Data_Sheet_1.docx]

Supplementary Material

Supplementary Table 1. Information on samples used in the study including taxon name, country for wild collections or cultivated, collection date, collector, herbarium information if sample is from a herbaria (Missouri Botanic Gardens (MOBOT), Australian National Herbarium (CANB), Queensland Herbarium (QLD)), id number, longitude, latitude and native or non-native status of sample in its collected location. All information has been included where available.

**Supplementary Table 2.** Genes and model of evolution used for analysis.

**Supplementary Figure 1.** Phylogenetic tree of samples in the foetida dataset (all samples of *Passiflora foetida* plus *Passiflora ambigua* as an outgroup) based on the bayesian analysis, shown with posterior probabilities values. The samples from Australia are coloured in khaki.

**Supplementary Figure 2.** Phylogenetic tree of samples in the Passifloraceae dataset based on the maximum likelihood analysis, shown with bootstrap confidence values. Samples of the native *Passiflora* species from Australia are coloured in khaki, the non-native naturalised and cultivated species in Australia in purple, and *Passiflora foetida sensu lato* in teal. Samples used as outgroups across analyses are in black.
